# Hemoglobin Concentration and the Incidence of Stroke in the General Japanese Population: The Jichi Medical School Cohort Study

**DOI:** 10.2188/jea.JE20200346

**Published:** 2022-03-05

**Authors:** Fumitaka Sato, Yosikazu Nakamura, Kazunori Kayaba, Shizukiyo Ishikawa

**Affiliations:** 1 Center for Community Medicine, Jichi Medical University, Tochigi, Japan; 2 Graduate School of Saitama Prefectural University, Saitama, Japan

**Keywords:** hemoglobin, stroke, subarachnoid hemorrhage, cohort study, Japanese population

## Abstract

**Background:**

Several studies have described an association between hemoglobin concentration and stroke; however, the influence of hemoglobin on stroke incidence has not been fully revealed. Our objective was to elucidate the association between hemoglobin concentration and stroke incidence in Japanese community residents.

**Methods:**

In the present study, we collected the data of 12,490 subjects who were enrolled between April 1992 and July 1995 in the Jichi Medical School (JMS) Cohort Study. We excluded the subjects with a history of stroke. Hemoglobin concentrations were grouped in quartiles, and quartile 2 (Q2) was used as the reference category. A Cox proportional-hazards model was used to examine hazard ratios (HRs) and the stroke incidence rates with 95% confidence intervals (CIs).

**Results:**

During 10.8 years of follow-up, 409 participants (212 men and 197 women) experienced a new stroke, including 97 intracerebral hemorrhages, 259 cerebral infarctions, and 52 subarachnoid hemorrhages (SAH). In sex-specific hemoglobin quartiles, the multivariate-adjusted HR was statistically significantly higher in Q1 than in Q2, and a relationship similar to a J shape was observed between all strokes (HR in Q2 vs Q1, 1.36; 95% CI, 1.02–1.83; Q3, 1.20; 95% CI, 0.87–1.64; and Q4, 1.16; 95% CI, 0.84–1.60). Furthermore, the analysis of stroke subtypes showed a statistically significantly higher multivariate-adjusted HR in Q1 than in Q2 for SAH (HR 2.61; 95% CI, 1.08–6.27).

**Conclusions:**

A low hemoglobin concentration was associated with an increased risk of stroke, which was strongly influenced by the incidence of SAH.

## INTRODUCTION

Stroke brings many deaths and long-term disability, which is an important issue for the world. In 2016, over 5 million deaths occurred and over 100 million disability-adjusted life-years were reported lost owing to stroke.^1^ Thus, it becomes imperative to identify predictors for the effective prevention of stroke. Previous studies showed a relationship between hematocrit or hemoglobin concentration and increased stroke onset.^2^^–^^10^ Wood et al described that increased hematocrit was associated with increased blood viscosity and peripheral resistance, which reduced cerebral circulation.^11^ However, the results of the previous studies were inconsistent regarding gender differences and degree of influence of hemoglobin on stroke onset. Several studies have pointed to the association between anemia and the incidence of stroke, but there was insufficient evidence due to relatively few stroke events.^2^^–^^4^^,^^8^^,^^9^

Though mortality rate associated with cardiovascular diseases is lower, its association with stroke is higher in Japan compared to the Western European countries.^12^ Considering the significant burden on the healthcare system and an increase in social security costs associated with strokes in Japan,^13^ observations on stroke incidence and mortality in the Japanese population is important. Most of the previous studies exploring the relationship between hemoglobin and stroke incidence were performed in the Western countries, and only a few studies have accounted for the relationship between the two in the Japanese population. The purpose of this study was to clarify the relation between hemoglobin concentration and stroke incidence in Japanese community residents.

## METHODS

### Study population

The JMS Cohort Study was conducted to investigate the factors responsible for cardiovascular and cerebrovascular disease in Japan.^14^ Data collection was carried out in 12 regions of Japan during the period April 1992 to July 1995 using a national mass screening system. There were 12,490 subjects (4,911 men, 7,579 women) who participated, who were 40–69 years of age in eight districts, ≥35 years of age in one district, and ≥18 years of age in three districts. Patients who have had a previous stroke were excluded.

We used the certificate from the public health center after receiving approval from the Agency of General Affairs and the Ministry of Health, Labour and Welfare.

### Questionnaire and measurements

Blood samples were obtained during the mass screening health check. Red blood cell count, hemoglobin, and hematocrit were measured in each laboratory in the districts. Serum total cholesterol (TC), high-density lipoprotein cholesterol (HDL-C), triglyceride (TG), and blood glucose (BG) concentrations were measured based on the use of enzymes, as previously published.^14^ Fibrinogen concentration was measured with a one-stage clotting assay kit (Data-Fi; Dade, Miami, FL, USA; inter-assay coefficient of variation = 2.5%). Serum high-sensitivity C-reactive protein (hsCRP) concentration was measured using highly sensitive nephelometry, a latex particle-enhanced immunoassay (NA Latex CRP Kit; Dade Behring, Tokyo, Japan). The internationally standardized hsCRP assay^15^ was sensitive enough to detect 0.030 mg/L of hsCRP, and the hsCRP value below the detection sensitivity was recorded as 0.015 mg/L.

Data on baseline medical history and lifestyle were gathered through patient-completed questionnaires. Systolic blood pressure (SBP) and diastolic blood pressure (DBP) were measured once in a sitting position after more than 5 minutes of rest, using an automatic blood pressure monitor (BP203RV-II; Nippon Colin, Komaki, Japan). Body mass index (BMI) was computed as weight in kilograms divided by square of height in meters. Smoking habit was categorized as current smoker or not, and alcohol drinkers were divided into drinking ≥20 g alcohol daily or not. Hypertension was defined as SBP/DBP ≥140/90 mm Hg and/or use of antihypertensive agents. Diabetes mellitus was defined as fasting BG ≥7.0 mmol/L (126 mg/dL), non-fasting BG ≥11.1 mmol/L (200 mg/dL) or use of oral hypoglycemic drugs or insulin. Hyperlipidemia was defined as TC ≥5.7 mmol/L (220 mg/dL) or the use of hypolipidemic agents according to the Japanese Atherosclerosis Society Guidelines for Prevention of Atherosclerotic Cardiovascular Diseases.^16^ The electrocardiogram (ECG) was recorded using ECG devices at each institution with a paper speed of 25 mm/s and a gain of 10 mm/mV (or 5 mm/mV). A diagnosis of left ventricular hypertrophy (LVH) was determined according to Cornell LVH criteria.^17^ Atrial fibrillation (AF) was independently determined by two cardiologists who reviewed the baseline ECG, and if the diagnoses were inconsistent, the final diagnosis was done after consideration by the approval committee.

### Assessment of outcomes

Most subjects were followed up annually until 2005 through a national mass screening program. Subjects who were examined were asked as to whether they experienced stroke or coronary heart disease, as reported previously.^18^ Subjects who did not attend the follow-up examinations were contacted by mail or telephone. Patients with such a history were inquired to provide the name of the hospital they visited, and their reason for admission was confirmed in medical records. Stroke was confirmed from the collected computed tomography information or magnetic resonance images, and myocardial infarction was confirmed from the collected electrocardiograms. In cases of confirmed stroke, medical records, computed tomography, and magnetic resonance imaging were collected from the hospital where the patients were examined. Diagnoses were made by a diagnosis committee, consisting of one radiologist, one neurologist, and two cardiologists. Stroke was defined as a focal and nonconvulsive neurological deficit with sudden onset and lasting at least 24 hours. At the time of the mass screening medical checkup, all respondents gave their individual written informed consent. This study design and methods were approved by each community government and the Institutional Review Board of JMS.

### Statistical analysis

Descriptive statistics were used to calculate quartile range of baseline hemoglobin. Statistical analyses were performed by sex-specific hemoglobin quartiles. Continuous variables we summarized as mean (standard deviation [SD]), and dichotomous variables (smoking status, alcohol drinking status, fasting state, diabetes mellitus, LVH, and AF) as percentages. To clarify the relationship between hemoglobin concentration and confounding factors, data were analyzed using one-way analysis of variance tests to evaluate overall gaps in continuous variable. Tukey’s honestly significant difference test was performed for comparison of means among four groups. The χ2 test was used to compare dichotomous variables. The risk of stroke incidence in relation to the hemoglobin concentration was expressed as hazard ratios (HRs) and 95% confidence intervals (CIs), calculated using the Cox-proportional hazards model. HRs and 95% CIs were calculated by adjusting for age and sex (HR1) and for age, sex, BMI, SBP, hypertension, diabetes mellitus, hyperlipidemia, smoking, and drinking status (HR2). The previous studies showed a J- or U-shaped curve between hemoglobin and risk of stroke, so quartile 2 (Q2) was selected as the control group.^2^^–^^4^ The crude stroke incidence was calculated per 1,000 person-years. We considered *P* values less than 0.05 to be statistically significant. All data analyses were conducted with SPSS^®^ (Version 26.0; IBM Corp, Armonk, NY, USA).

## RESULTS

The baseline and follow-up data of subjects by the quartiles of baseline hemoglobin are shown for both the sexes in Table 1. Baseline hemoglobin positively correlated with BMI, TC, and TG in both sexes. In men, baseline hemoglobin positively correlated with current drinking and negatively correlated with HDL-C, fibrinogen, and hsCRP. In women, baseline hemoglobin showed a positive correlation with SBP and presence of diabetes mellitus and LVH.

**Table 1.** Baseline characteristics of study participants by quartiles of hemoglobin for both the sexes

	Hemoglobin, mg/dL				
	
Men	Q1 (≦14.2 mg/dL)	Q2 (14.3–15.1 mg/dL)	Q3 (15.2–15.8 mg/dL)	Q4 (>15.8 mg/dL)	*P*
Subject, *n*	1,254	1,136	1,040	1,131	
Age, years	59.8 (10.4)	55.9 (11.2)	52.9 (12.2)	51.3 (11.6)	<0.001^a^
Body mass index, kg/m^2^	21.9 (2.7)	22.9 (2.7)	23.2 (2.8)	24.1 (3.0)	<0.001^a^
Systolic blood pressure, mm Hg	129.1 (20.4)	130.5 (20.5)	130.4 (19.3)	135.8 (20.8)	<0.001^a^
Diastolic blood pressure, mm Hg	77.3 (12.3)	78.3 (11.9)	78.7 (11.6)	82.7 (12.4)	<0.001^a^
Total cholesterol, mg/dL	175.3 (32.9)	184.6 (31.7)	187.8 (32.4)	194.7 (35.8)	<0.001^a^
HDL cholesterol, mg/dL	49.9 (13.4)	49.8 (12.9)	49.0 (13.5)	46.5 (12.9)	<0.001^a^
Triglycerides, mg/dL	104.4 (61.8)	123.9 (76.7)	128.0 (80.7)	159.8 (112.1)	<0.001^a^
Blood glucose, mg/dL	108.7 (30.4)	106.7 (34.4)	103.8 (29.3)	105.1 (31.4)	0.001^a^
Hematocrit, %	40.8 (2.7)	44.1 (1.4)	46.0 (1.5)	48.8 (2.3)	<0.001^a^
Fibrinogen, mg/dL	251.0 (60.4)	246.0 (58.6)	240.6 (55.3)	231.8 (49.8)	<0.001^a^
hsCRP, mg/L	1,427.8 (5,684.8)	919.9 (4,323.7)	707.9 (2,936.2)	699.4 (2,579.0)	0.005^a^
Current smoking, %	46.3	46.0	50.2	59.2	<0.001^b^
Current alcohol drinking, %	73.6	75.3	76.0	77.7	0.013^b^
Fasting state, %	47.2	50.1	56.6	64.8	<0.001^b^
Diabetes mellitus, %	5.3	5.0	5.0	5.7	0.93^b^
Left ventricular hypertrophy, %	2.8	2.8	3.1	3.3	<0.001^b^
Atrial fibrillation, %	0.6	0.7	0.5	0.7	<0.001^b^

Women	Q1 (≦12.4 mg/dL)	Q2 (12.5–13.1 mg/dL)	Q3 (13.2–13.8 mg/dL)	Q4 (>13.8 mg/dL)	*P*

Subject, *n*	1,931	1,780	1,762	1,676	
Age, years	54.1 (11.9)	55.0 (11.2)	56.0 (10.9)	55.9 (10.3)	<0.001^a^
Body mass index, kg/m^2^	22.4 (3.1)	22.9 (3.0)	23.5 (3.2)	24.0 (3.4)	<0.001^a^
Systolic blood pressure, mm Hg	122.5 (19.9)	125.6 (19.9)	130.1 (20.6)	134.7 (21.0)	<0.001^a^
Diastolic blood pressure, mm Hg	72.8 (11.5)	74.8 (11.4)	77.7 (11.9)	80.3 (12.0)	<0.001^a^
Total cholesterol, mg/dL	187.3 (33.6)	195.1 (33.6)	199.7 (33.5)	206.7 (35.5)	<0.001^a^
HDL cholesterol, mg/dL	52.8 (12.5)	53.3 (12.6)	52.2 (12.0)	52.1 (13.0)	0.019^a^
Triglycerides, mg/dL	97.6 (50.4)	105.9 (65.2)	117.8 (78.5)	121.4 (73.6)	<0.001^a^
Blood glucose, mg/dL	100.9 (20.8)	100.1 (20.2)	101.1 (22.7)	102.5 (27.4)	0.022^a^
Hematocrit, %	40.8 (2.7)	44.1 (1.4)	46.0 (1.5)	48.8 (2.3)	<0.001^a^
Fibrinogen, mg/dL	249.5 (59.1)	245.2 (52.3)	250.3 (53.9)	250.7 (54.2)	0.097^a^
hsCRP, mg/L	650.7 (2,577.2)	540.1 (2,922.0)	551.1 (2,846.6)	682.5 (3,123.5)	0.613^a^
Current smoking, %	5.4	5.1	4.2	7.4	0.005^b^
Current alcohol drinking, %	23.6	23.2	24.4	28.5	0.008^b^
Fasting state, %	43.6	49.4	52.8	63.8	<0.001^b^
Diabetes mellitus, %	1.6	1.9	3.2	4.2	<0.001^b^
Left ventricular hypertrophy, %	6.4	7.4	8.6	11.3	<0.001^b^
Atrial fibrillation, %	0.2	0.2	0.5	0.4	0.496^b^

HDL, high-density lipoprotein; hsCRP, High-sensitivity C-reactive protein; Q, quartile.

^a^One-way analysis of variance test; ^b^χ2 test.

Data are expressed as mean (standard deviation) for variables and as percentage for rates.

During the 10.8 years of follow-up, 409 subjects (212 men and 197 women) developed a new stroke. There were 97 intracerebral hemorrhages (48 in men and 49 in women), 259 cerebral infarctions (152 in men and 107 in women), and 52 subarachnoid hemorrhages (SAH) (12 in men and 40 in women) reported.

Table 2 lists the incidence rates of stroke and adjusted HRs with 95% CI by categories using sex-specific hemoglobin quartiles. HR2 was statistically significantly higher in Q1 than in Q2 (control group), and a relationship similar to a J shape was observed between all strokes (HR2s in Q2 vs Q1, 1.36; 95% CI, 1.02–1.83; Q2 vs Q3, 1.20; 95% CI, 0.87–1.64; and Q2 vs Q4, 1.16; 95% CI, 0.84–1.60). HR2 for intracerebral hemorrhage and cerebral infarction were higher in Q1 than in Q2, but not statistically significant. HR2 in Q1 for SAH was statistically significantly higher than that in Q2 (HR2 2.61; 95% CI, 1.08–6.27).

**Table 2.** Hazard ratios based on total quartiles of sex-specific hemoglobin and adjusted for potential confounders

	Hemoglobin, mg/dL			

	Q1 (Men: ≦14.2 mg/dL, Women: ≦12.4 mg/dL)	Q2 (Men: 14.3–15.1 mg/dL, Women: 12.5–13.1 mg/dL)	Q3 (Men: 15.2–15.8 mg/dL, Women: 13.2–13.8 mg/dL)	Q4 (Men: >15.8 mg/dL, Women: >13.8 mg/dL)
All-stroke				
Number of cases	135	82	95	97
Incidence rate^a^	406	262	313	322
HR1 (95% CI)	1.34 (1.01–1.76)	1.00	1.27 (0.95–1.71)	1.45 (1.08–1.95)
HR2 (95% CI)	1.36 (1.02–1.83)	1.00	1.20 (0.87–1.64)	1.16 (0.84–1.60)
Intracerebral hemorrhage				
Number of cases	32	17	27	21
Incidence rate^a^	96	54	89	70
HR1 (95% CI)	1.56 (0.86–2.81)	1.00	1.73 (0.94–3.17)	1.49 (0.78–2.83)
HR2 (95% CI)	1.34 (0.73–2.47)	1.00	1.43 (0.76–2.69)	0.94 (0.46–1.91)
Cerebral infraction				
Number of cases	84	57	52	66
Incidence rate^a^	252	182	172	219
HR1 (95% CI)	1.14 (0.82–1.61)	1.00	1.03 (0.71–1.50)	1.49 (1.04–2.13)
HR2 (95% CI)	1.18 (0.82–1.70)	1.00	1.01 (0.67–1.51)	1.26 (0.85–1.82)
Subarachnoid hemorrhage				
Number of cases	18	8	16	10
Incidence rate^a^	54	26	53	33
HR1 (95% CI)	2.08 (0.90–4.77)	1.00	2.03 (0.87–4.74)	1.34 (0.53–3.39)
HR2 (95% CI)	2.61 (1.08–6.27)	1.00	1.93 (0.78–4.80)	1.19 (0.44–3.17)

CI, confidence interval; HR, hazard ratio.

HR1: Hazard ratios adjusted for age and sex.

HR2: Hazard ratios adjusted for age, sex, systolic blood pressure, total cholesterol, high-density lipoprotein cholesterol, triglycerides, diabetes mellitus, smoking, and alcohol consumption.

^a^Per 100,000 person-years.

Adjusted HRs with 95% CI by sexes are shown in Table 3. The incidence of all strokes was higher in men than in women in all hemoglobin quartiles. The incidence of intracerebral hemorrhage and cerebral infarction were higher in men than in women. However, the incidence of SAH was higher in women than in men in each hemoglobin quartile. HR2 for all-stroke cases exhibited a curve similar to a J shape in men, whereas HR2 in Q3 and Q4 were similar to HR2 in Q2 in women. HR2 for SAH was 5.8 times higher in Q1 than in Q2 in men (HR2, 5.87; 95% CI, 0.69–50.22), and 2.0 times higher in Q1 than in Q2 in women (HR2, 2.07; 95% CI, 0.77–5.33). However, the difference was not statistically significant. In both sexes, no other statistically significant differences between hemoglobin quartiles and any stroke-type specific incidents were found.

**Table 3.** Hazard ratios based on Hemoglobin and adjusted for potential confounders for both the sexes

	Hemoglobin, mg/dL			
	
Men	Q1 (≦14.2 mg/dL)	Q2 (14.3–15.1 mg/dL)	Q3 (15.2–15.8 mg/dL)	Q4 (>15.8 mg/dL)
All-stroke				
Number of cases	76	42	47	47
Incidence rate^a^	596	350	421	394
HR1 (95% CI)	1.31 (0.90–1.91)	1.00	1.44 (0.95–2.18)	1.58 (1.04–2.40)
HR2 (95% CI)	1.34 (0.89–2.01)	1.00	1.52 (0.98–2.35)	1.31 (0.83–2.08)
Intracerebral hemorrhage				
Number of cases	17	8	11	12
Incidence rate^a^	133	67	99	101
HR1 (95% CI)	1.58 (0.68–3.68)	1.00	1.73 (0.69–4.30)	2.03 (0.83–4.99)
HR2 (95% CI)	1.26 (0.52–3.04)	1.00	1.65 (0.63–4.34)	1.47 (0.54–4.04)
Cerebral infraction				
Number of cases	53	33	33	33
Incidence rate^a^	415	275	296	276
HR1 (95% CI)	1.14 (0.74–1.77)	1.00	1.30 (0.80–2.11)	1.45 (0.89–2.35)
HR2 (95% CI)	1.22 (0.76–1.96)	1.00	1.46 (0.88–2.42)	1.26 (0.74–2.15)
Subarachnoid hemorrhage				
Number of cases	6	1	3	2
Incidence rate^a^	47	8	27	17
HR1 (95% CI)	4.78 (0.57–40.01)	1.00	3.62 (0.38–34.92)	2.45 (0.22–27.30)
HR2 (95% CI)	5.87 (0.69–50.22)	1.00	2.31 (0.21–25.63)	2.01 (0.17–23.09)

Women	Q1 (≦12.4 mg/dL)	Q2 (12.5–13.1 mg/dL)	Q3 (13.2–13.8 mg/dL)	Q4 (>13.8 mg/dL)

All-stroke				
Number of cases	59	40	48	50
Incidence rate^a^	287	208	251	276
HR1 (95% CI)	1.39 (0.93–2.08)	1.00	1.11 (0.73–1.70)	1.32 (0.87–2.00)
HR2 (95% CI)	1.4 (0.91–2.15)	1.00	0.95 (0.60–1.49)	1.05 (0.66–1.65)
Intracerebral hemorrhage				
Number of cases	15	9	16	9
Incidence rate^a^	73	47	84	50
HR1 (95% CI)	1.58 (0.69–3.61)	1.00	1.65 (0.73–3.73)	1.05 (0.42–2.65)
HR2 (95% CI)	1.39 (0.59–3.28)	1.00	1.39 (0.60–3.22)	0.70 (0.26–1.93)
Cerebral infraction				
Number of cases	31	24	19	33
Incidence rate^a^	151	125	99	182
HR1 (95% CI)	1.18 (0.69–2.02)	1.00	0.72 (0.39–1.31)	1.48 (0.87–2.50)
HR2 (95% CI)	1.14 (0.64–2.03)	1.00	0.51 (0.26–1.03)	1.20 (0.67–2.16)
Subarachnoid hemorrhage				
Number of cases	12	7	13	8
Incidence rate^a^	58	36	68	44
HR1 (95% CI)	1.64 (0.65–4.18)	1.00	1.80 (0.72–4.51)	1.19 (0.43–3.27)
HR2 (95% CI)	2.07 (0.77–5.53)	1.00	1.81 (0.68–4.86)	1.04 (0.35–3.06)

CI, confidence interval; HR, hazard ratio.

HR1: Hazard ratios adjusted for age.

HR2: Hazard ratios adjusted for age, systolic blood pressure, total cholesterol, high-density lipoprotein cholesterol, triglycerides, diabetes mellitus, smoking, and alcohol consumption.

^a^Per 100,000 person-years.

## DISCUSSION

In this cohort study, a relationship similar to a J shape was observed between hemoglobin concentration and risk of stroke. This correlation was mainly found among men. Among stroke subtypes, a low hemoglobin concentration strongly influenced the incidence of SAH. These findings indicate that hemoglobin concentration can be used as a predictor to assess the risk of stroke.

Previous studies have shown that hematocrit or hemoglobin concentration related to increased stroke onset; however, the observations on the degree of influence of hemoglobin on stroke incidence and the impact of gender on their relationship was inconsistent. High levels of hemoglobin were related to stroke risk in the Framingham cohort study,^5^ and high levels of hematocrit were an independent risk of stroke onset in the British Regional Heart Study.^7^ The Hisayama cohort study showed a U-shaped curve between hematocrit levels and incidence of ischemic stroke, and hematocrit levels and hemorrhagic stroke were linearly inversely related.^4^ A U-shaped curve was also observed between male hemoglobin levels and hemorrhagic stroke in South Korea.^8^ Several studies showed the association between low hemoglobin concentration and hemorrhagic stroke, which were similar to our results. Our important findings revealed that a low hemoglobin concentration was significantly associated with an increased risk of SAH incidence. There are no previous reports directly investigating the association between low hemoglobin concentration and incidence of SAH.

Of all the underlying causes of SAH, 85% of those excluding traumatic injuries are attributed to ruptured cerebral aneurysms.^19^ Saccular aneurysms represent local structural deterioration of the arterial wall, such as internal elastic lamina deficiency and media disruption,^20^ and exhibit the prominent features of inflammation and tissue degeneration. Hemodynamic stress and inflammatory responses were speculated to have key functions in the deterioration of the extracellular matrix and apoptosis of smooth muscle cells during the formation of aneurysms.^21^ Inflammation due to the cigarette smoke also has a strong effect on the formation, progression, and rupture of cerebral aneurysms.^22^ A study on the relationship between ruptured cerebral aneurysms and anemia reported that the patients with ruptured cerebral aneurysms were more anemic and had lower total iron-binding capacity (TIBC), which was assumed to reflect chronic inflammatory conditions.^23^ In the present study, high concentrations of serum fibrinogen and hsCRP, known inflammatory markers, correlated with low hemoglobin concentrations in men (Table 1). If an anemia condition suggests a chronic inflammatory condition, it may explain SAH prevalence in the anemic group in our study. However, the role of inflammation as the underlying mechanism linking low hemoglobin concentrations and the occurrence of SAH is a hypothesis and requires further validation.

The association between hemoglobin concentration and increased stroke onset has been explained by various pathophysiological mechanisms. High levels of hemoglobin have been reported to lead to an increase in blood viscosity and a decrease in cerebral circulation, which may increase the risk of stroke.^11^ Similarly, anemia can also have a negative effect on the cerebrovascular system. Anemia causes ventricular remodeling and cardiac dysfunction, resulting in increased cardiac output^24^ and LVH, which ultimately contributes to an increased risk of stroke,^25^ and the effect of anemia on stroke has also been reported in the context of chronic kidney disease.^26^^,^^27^ Table 2 shows the statistically significant relationship between HR2 in Q1 and all stroke onset, whereas the same was not valid for the relationship between HR2 in Q4 and all stroke onset. To further assess the association between high hemoglobin concentration and stroke onset, we used sex-specific hemoglobin measurements to both determine the top decile of the hemoglobin group and calculate HRs for stroke onset. In the top decile for all strokes, HR1 and HR2 were 1.58 (95% CI, 1.09–2.29) and 1.18 (95% CI, 0.79–1.78), respectively. Furthermore, an increase in risk was not observed. Although high hemoglobin concentration is considered one of the risk factors for stroke and a low hemoglobin concentration is regarded to be an excellent marker associated with the chronic diseases that predispose the patients to stroke, several unclear points that need further investigation remain.

It is still unknown how sex impacts the relation between hemoglobin and stroke risk. The incidence of intracerebral hemorrhage and cerebral infarction was higher in men than in women, whereas the incidence of SAH was higher in women than in men when compared by each hemoglobin quartile in the present study. Women are generally more likely to have SAH than men, and it has been suggested that a deficiency of estrogen is associated with higher risk of aneurysm formation.^28^ Despite women having lower mean hemoglobin concentrations than men, low hemoglobin concentrations were only associated with risk of SAH in men. The contribution of hemoglobin concentration to SAH may be weak in most women.

### Limitations

Our study includes several potential limitations. First, subjects in our study were enrolled from a general medical checkup, but the selection was not randomized and may not reflect the general Japanese population. Because of the voluntary participation in a health examination, the proportion of those receiving treatment for hypertension, diabetes, and dyslipidemia were lower than that reported in national health surveys.^29^ Second, since baseline medical history and lifestyle information was obtained through questionnaires, all the information regarding smoking, drinking, and medication history were self-reported and some inaccuracies are expected. Third, the incidence of stroke may be underestimated, as it does not include cases involving asymptomatic stroke. Fourth, our research design is an observational study, so causal relationships remain unanswered. Finally, since hemoglobin was measured once, it is not possible to decide whether long-term variations in hemoglobin have any relation with stroke risk. The strength of the present study is the large size of the study sample in the Japanese population and the fact that stroke diagnoses were made within a long follow-up period. This result can be clinically important, especially for the Japanese population.

In conclusion, our study showed a relationship similar to a J shape between hemoglobin concentration and the risk of stroke. Furthermore, a low hemoglobin concentration was associated with an increased risk of stroke, which was most strongly influenced by the incidence of SAH. These findings may provide valuable information on the association between hemoglobin and stroke incidence.
